# Printed Piezoelectric Materials: From Functional Inks to High-Performance Transducers

**DOI:** 10.3390/s26102961

**Published:** 2026-05-08

**Authors:** Manuel Reis Carneiro

**Affiliations:** Department of Health Sciences and Technology, ETH Zürich, 8008 Zürich, Switzerland; manuel.reiscarneiro@hest.ethz.ch

**Keywords:** piezoelectric inks, piezoelectric transducers, printed electronics, additive manufacturing, printed transducers, ceramic–polymer hybrids, low-temperature processing, direct ink writing, stereolithography

## Abstract

Printable piezoelectric materials are emerging as a cornerstone of next-generation sensing, actuation, and energy harvesting technologies, driven by the need for lightweight, flexible, and digitally manufactured transducers. Conventional ceramic piezoelectrics offer exceptional electromechanical performance but require high-temperature sintering and exhibit intrinsic brittleness, limiting their integration with soft or unconventional substrates. Polymeric piezoelectrics, in contrast, provide mechanical compliance and low-temperature processability yet suffer from lower crystallinity, reduced piezoelectric coefficients, and limited thermal stability. These contrasting characteristics have catalyzed the development of functional piezoelectric inks—ceramic, polymeric, and hybrid formulations engineered for additive manufacturing techniques such as direct ink writing, stereolithography, screen printing, and inkjet printing. This review systematically examines the material compositions, dispersion chemistries, printing requirements, thermal treatment pathways, and poling strategies that govern the performance of printed piezoelectric transducers. By comparing ceramic-based, polymer-based, and hybrid systems, we reveal the fundamental trade-offs between printability, crystallinity, mechanical compliance, and electromechanical response, and map how these trade-offs shape device design across wearable electronics, soft robotics, and structural health monitoring. Finally, we highlight emerging approaches—including surface functionalization, low-temperature crystallization, liquid-phase sintering, and engineered ceramic–polymer interfaces—that offer promising routes to bridge the gap between printability and high piezoelectric performance.

## 1. Introduction

Piezoelectric materials convert mechanical stimuli into electrical signals and vice versa, enabling applications in sensing [1,2,3,4], actuation [4,5], energy harvesting [1,6], and biomedical devices [6,7]. Conventional piezoelectric ceramics offer high electromechanical performance but remain rigid, brittle, and incompatible with flexible substrates or digital manufacturing workflows. Polymeric piezoelectrics provide mechanical compliance but typically exhibit lower piezoelectric coefficients and limited thermal stability. These limitations have intensified the search for materials that combine high piezoelectric performance with mechanical flexibility and compatibility with modern additive manufacturing [8,9,10,11,12,13,14].

Over the past decade, advances in printed electronics have demonstrated that functional materials can be patterned directly onto soft, curved, or textile substrates, enabling new classes of wearable and conformable devices [15,16,17,18]. Printed systems for AI and health monitoring [19,20,21,22,23,24,25,26,27,28,29,30,31,32], motion tracking [33,34,35,36,37,38,39,40,41,42], and even novel actuation [43,44] or energy storage [45,46] strategies have shown how additive manufacturing can deliver thin, lightweight, and body-conforming interfaces with minimal fabrication complexity. These developments highlight a broader trend: printing is becoming a foundational strategy for creating unobtrusive, customizable, and scalable wearable technologies.

Within this context, printable piezoelectric inks represent a critical next step. By formulating piezo-active ceramics, polymers, or hybrid mixtures into inks compatible with techniques such as direct ink writing, screen printing, stereolithography, and inkjet printing, researchers can fabricate piezoelectric transducers with complex geometries, tunable thicknesses, and seamless integration onto flexible or unconventional substrates. This shift from bulk processing to digitally patterned piezoelectric functionality opens new opportunities in soft robotics, structural health monitoring, and wearable sensing—particularly where conformability, low weight, and distributed sensing architectures are required.

**Figure 1:**
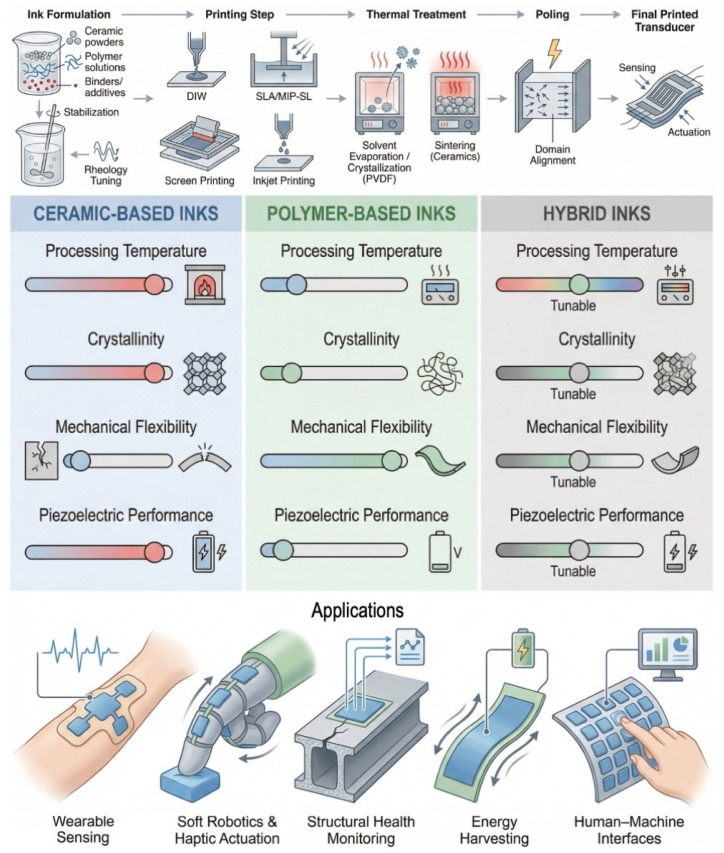
Overview of the processing workflow, material characteristics, and application domains of printed piezoelectric transducers. **Fabrication pipeline** (**top panel**): Ink formulation, printing (e.g., DIW, screen printing, inkjet printing), thermal treatment (solvent evaporation, pyrolysis, sintering), and poling steps leading to functional piezoelectric devices. **Material classes and trade-offs** (**middle panel**): Comparison of ceramic-based, polymer-based, and hybrid inks, highlighting differences in processing temperature, crystallinity, mechanical flexibility, and piezoelectric performance. **Application domains** (**bottom panel**): Representative use cases enabled by printed piezoelectric materials, including wearable sensing, soft robotics and haptic actuation, structural health monitoring, energy harvesting, and human–machine interfaces.

This review examines the current landscape of functional piezoelectric inks and printable piezoelectric compounds, focusing on their material compositions, synthesis strategies, printing methods, and resulting electromechanical performance. By comparing ceramic-based, polymer-based, and hybrid formulations, as shown in Figure 1, we highlight the fundamental trade-offs between printability, crystallinity, mechanical compliance, and piezoelectric response. We further discuss how these trade-offs shape the design of printed transducers for emerging applications and identify promising research directions—including surface functionalization, low-temperature crystallization routes, and ceramic–polymer hybrid architectures—that may bridge the gap between printability and performance.

While previous reviews survey printable piezoelectric materials or summarize individual printing techniques, these works typically treat materials, processing, and device performance as separate topics. What is missing is an integrated “ink-to-transducer” perspective that connects ink formulation, printability constraints, thermal and poling requirements, and the resulting electromechanical behavior of the printed device. The present review fills this gap by comparing ceramic, polymer, and hybrid inks within a unified processing–structure–property framework and by mapping how printing-specific parameters—rheology, curing pathways, densification, crystallinity, and interfacial engineering—govern the performance of printed transducers. This approach provides a consolidated view that is not available in prior reviews and highlights the design principles that enable high-performance piezoelectric devices to be realized through additive manufacturing.

## 2. Implementation of Printed Piezoelectric Transducers

**Figure 2:**
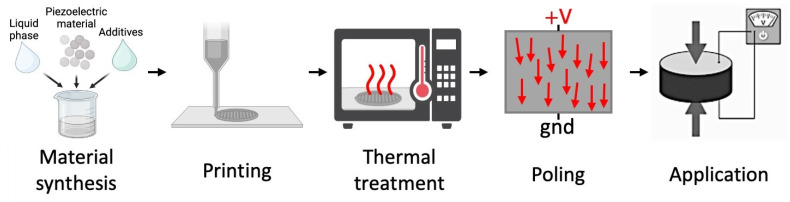
Implementation pipeline for printed piezoelectric transducers.

The fabrication of printed piezoelectric transducers, regardless of the specific material system, follows a broadly consistent pipeline comprising ink formulation, printing, thermal treatment, and polarization (poling). Each stage plays a decisive role in defining the microstructure, electromechanical response, and long-term stability of the final device. The overall workflow is illustrated in Figure 2.

### 2.1. Thermal Treatment

Thermal treatment is a critical step for both polymer-based and ceramic-based printable piezoelectric compounds, but the underlying mechanisms and temperature requirements differ substantially.

For materials such as PVDF and its copolymers, thermal treatment primarily removes residual solvents and promotes chain mobility, enabling crystallization into electroactive phases. Annealing near the melting point of PVDF (~150 °C) is commonly used to enhance β-phase content and improve dielectric and piezoelectric properties [47,48,49,50]. The thermal window is narrow: insufficient heating limits crystallinity, while excessive heating can degrade the polymer or disrupt printed geometries.

Printable ceramic slurries—typically based on PZT, BaTiO_3_, BCZT, or KNN, as shown in further sections—require, on the other hand, high-temperature sintering to densify the structure, eliminate organic binders, and promote grain growth [51,52,53]. Sintering temperatures often exceed 1000 °C, with the exact profile tailored to the ceramic composition and particle size distribution. Achieving high-density and well-defined crystalline phases is essential for maximizing the piezoelectric charge coefficient and mechanical robustness [54,55,56,57,58].

Across both material classes, the thermal treatment step governs crystallinity, density, phase purity, and mechanical integrity, making it one of the most influential parameters in the entire fabrication pipeline.

### 2.2. Poling

Following thermal treatment, the dipoles within the piezoelectric material remain randomly oriented, resulting in negligible net polarization. Poling is therefore required to activate the material.

During poling, a high DC electric field is applied across the printed structure for a defined duration, often at an elevated temperature. The field aligns the ferroelectric domains along its direction, establishing a remanent polarization that enables both sensing (direct effect) and actuation (inverse effect). Typical poling fields range from hundreds of volts per micrometer in polymers to several kilovolts per millimeter in ceramics, constrained by the dielectric breakdown strength of the material [48,59,60].

Temperature in the poling process plays a dual role: it increases dipole mobility—particularly important for polymers such as PVDF—yet must remain below the Curie temperature to avoid depolarization. Optimizing the interplay between field strength, temperature, and time is essential for maximizing the piezoelectric response without compromising structural integrity.

### 2.3. Interdependence of Processing Steps

Thermal treatment and poling are deeply interconnected. The degree of crystallinity, grain size, and density established during thermal processing directly influence the efficiency of domain alignment during poling. Conversely, suboptimal poling can mask the intrinsic performance of a well-processed material. Together, these steps determine the final piezoelectric coefficient, dielectric behavior, mechanical strength, and long-term stability of printed transducers.

## 3. Printable Piezoelectric Material Architectures

Printable piezoelectric materials have evolved along three main directions: ceramic-based formulations, polymer-based systems, and hybrid mixtures that attempt to combine the strengths of both. Ceramic-based inks remain the most widely explored due to their superior electromechanical performance, and they effectively illustrate the interplay between dispersion chemistry, printing strategy, thermal processing, and poling conditions. The following narrative traces how different research groups have approached the challenge of translating high-performance piezoelectric ceramics into printable, shape-programmable architectures.

### 3.1. Ceramic-Based Compounds

Among all piezoelectric ceramics, lead zirconate titanate (PZT) continues to serve as the reference material because of its high piezoelectric coefficients and well-established processing routes. Unsurprisingly, many of the earliest and most successful printable piezoelectric inks are based on PZT powders dispersed in carefully engineered solvent–binder systems. In one representative example, Liu and colleagues [61] dispersed PZT in ethanol using trisodium citrate to suppress agglomeration, then incorporated the stabilized particles into a PVS–water matrix to obtain a direct ink writing slurry. After printing, the organic phase was removed at 600 °C and the ceramic was sintered at 1250 °C for several hours to promote grain growth and densification. The printed structures were subsequently infiltrated with epoxy to improve mechanical robustness, coated with silver electrodes, and poled at 2.5 kV/mm, ultimately achieving a piezoelectric coefficient of 103 pC/N. This workflow illustrates the classical DIW route: dispersion, extrusion, high-temperature sintering, and post-processing to compensate for brittleness.

A similar strategy was adopted by Li et al. [62], who used a xylene–ethanol solvent system with triethyl phosphate as a dispersant, followed by prolonged ball-milling to homogenize the suspension. Plasticizers and PVB binder were added to tune the rheology for DIW, and a two-step thermal treatment—first for binder burnout, then for sintering at 1250 °C—yielded dense PZT structures. After copper electrodes were attached and the samples were poled at 3.5 kV/mm in a heated oil bath, the resulting transducers reached 265 pC/N, demonstrating how optimized dispersion and sintering can substantially enhance performance.

More recently, researchers have explored stereolithography as a route to more intricate geometries. Lu et al. [63] introduced a liquid-phase sintering approach in which PZT powder was mixed with lead nitrate and a UV-curable resin. During thermal treatment, the lead nitrate decomposed into molten lead oxide, which acted as a transient liquid phase that pulled particles together through surface tension-driven rearrangement. This mechanism enabled densification at lower temperatures before final crystallization at 1100 °C. The printed and poled structures achieved a remarkably high d_33_ of 583 pC/N, underscoring the potential of liquid-phase sintering to overcome porosity and grain boundary limitations in printed ceramics.

Variants of PZT, such as lanthanum-modified PLZT, have also been adapted for printing. In work by Li et al. [64], PLZT powders were dispersed in water with sodium citrate and printed via DIW, followed by a multi-step thermal treatment in a lead-rich atmosphere to prevent volatilization. After epoxy infiltration and poling at 3.5 kV/mm, the printed PLZT structures reached 347 pC/N, showing that compositional modifications can be successfully transferred into printable architectures.

Environmental concerns surrounding lead have motivated the development of lead-free printable ceramics, though their performance generally remains below that of PZT. Several groups have explored barium zirconate titanate–barium calcium titanate (BCZT), barium titanate (BaTiO_3_), or potassium sodium niobate (KNN) as alternatives [65]. Nan et al. [66] formulated a BCZT-based DIW ink using a combination of dispersant, binder, and coagulating agent to stabilize the slurry. After printing and sintering at temperatures up to 1500 °C, the resulting structures achieved 100 pC/N. Li et al. [67] developed a photocurable KNN suspension that could be printed and sintered between 1050 and 1130 °C, yielding d_33_ values up to 280 pC/N—one of the highest reported for printed lead-free ceramics. BaTiO_3_-based systems have also been explored through stereolithography, as demonstrated by Chen et al. [68], who used deagglomerated nanoparticles in a photocurable resin. After debinding and sintering at 1330 °C, the printed structures reached 160 pC/N, while later work from the same group [69] showed that lower poling fields can significantly reduce performance. In contrast, Nguyen et al. [70] attempted to bypass high-temperature sintering by UV-curing a BaTiO_3_–PUA mixture, but the absence of ceramic densification resulted in a very low d_33_ of 1.31 pC/N, reinforcing the necessity of sintering for ceramic-based inks.

A particularly innovative direction involves bypassing high-temperature sintering altogether. Cui et al. [71] demonstrated that surface-functionalizing PZT particles with trimethoxysilyl propyl methacrylate enables strong interfacial bonding within a photocurable resin, allowing the fabrication of complex 3D structures via stereolithography without any thermal sintering. Although the resulting d_33_ of 100 pC/N remains below that of fully sintered ceramics, the ability to print piezoelectric structures at low temperatures represents a significant step toward integrating piezoelectric functionality into flexible substrates and multi-material additive manufacturing platforms.

Across these studies, a clear pattern emerges: ceramic-based printable inks can achieve high piezoelectric performance, but only when dispersion, printing, thermal treatment, and poling are carefully orchestrated. High-temperature sintering remains the dominant route to dense, high-performance ceramics, while emerging strategies such as liquid-phase sintering and surface functionalization offer promising pathways toward lower-temperature processing and more complex geometries. The tension between printability, processing temperature, and electromechanical performance defines the landscape of ceramic-based printable piezoelectric materials and sets the stage for hybrid and polymer-based approaches discussed in the following sections.

### 3.2. Polymer-Based Compounds

Polymer-based piezoelectric materials have attracted sustained interest because they offer a set of properties that ceramic systems fundamentally cannot provide: mechanical flexibility, low density, compatibility with large area processing, and the ability to conform to curved or deformable substrates. Among these materials, polyvinylidene fluoride (PVDF) and its copolymers—particularly P(VDF-TrFE)—have become the dominant choice for printable piezoelectric systems. Their piezoelectricity arises from the alignment of molecular dipoles within the β-phase crystalline structure, and much of the research in this area has focused on developing ink formulations and processing routes that maximize β-phase content while preserving printability.

Early work by Brown et al. [72] demonstrated the potential of P(VDF-TrFE) for printed piezoelectric films. Using a simple spin casting approach with methyl ethyl ketone as a solvent, the authors produced thin polymer layers that were annealed at 140 °C and poled under high electric fields. Although the piezoelectric coefficient was not reported, this study established the basic processing sequence—solvent casting, thermal treatment, and high field poling—that would underpin later printable formulations.

As printing technologies matured, researchers began adapting P(VDF-TrFE) to screen printing, inkjet printing, and stencil printing. Zirkl et al. [73] dissolved the copolymer in a γ butyrolactone/acetone mixture to obtain a screen-printable ink that could be cured at relatively low temperatures. After poling at 80 V/µm, the printed films achieved a d_33_ of 32 pC/N, illustrating that polymer-based inks can deliver moderate piezoelectric performance while remaining compatible with low-temperature substrates. Thuau et al. [74] pursued a similar strategy using a cyclopentanone/DMSO solvent system tailored for inkjet printing. Their printed films, cured at 140 °C and poled at 100 MV/m, reached 10.4 pC/N, highlighting how the choice of solvent, printing method, and poling conditions can strongly influence the final response.

Stencil printing has also been used to pattern P(VDF-TrFE) films. Rajala et al. [75] employed methyl ethyl ketone as a solvent and cured the printed layers at 120 °C before poling at 65 MV/m, obtaining a d_33_ of 22 pC/N. These studies collectively show that polymer-based inks can be processed at temperatures well below those required for ceramics, enabling integration with flexible substrates and multilayer printed electronics.

Environmental considerations have motivated the search for greener solvent systems. Gonçalves et al. [76] replaced the commonly used DMF with DMPU to formulate a more environmentally benign P(VDF-TrFE) paste. The ink could be patterned through multiple printing techniques, and after curing at 230 °C and poling at 1 MV/cm, the resulting films exhibited a d_33_ of 19 pC/N. This work demonstrated that sustainable solvent choices can be made without compromising printability or electromechanical performance.

Beyond solution-processed films, melt-based approaches have also been explored. Zhang et al. [77] used melt extrusion to cast PVDF films at 250 °C, followed by poling at extremely high electric fields (~240 kV/mm). The resulting pure PVDF films exhibited a modest d_33_ of 4 pC/N, reflecting the intrinsic limitations of the polymer. However, this study also laid the groundwork for hybrid polymer–ceramic systems, showing that the mechanical robustness and processability of PVDF can be combined with ceramic fillers to enhance piezoelectric performance—a direction further developed in the hybrid materials section.

Taken together, these studies illustrate the central trade-off of polymer-based printable piezoelectrics: they offer unmatched flexibility, low-temperature processing, and compatibility with wearable and conformable devices, but their piezoelectric coefficients remain significantly lower than those of ceramic-based inks. Nevertheless, their ease of printing, environmental adaptability, and mechanical compliance make them indispensable for applications where ceramics are unsuitable, and they form the foundation for hybrid approaches that seek to combine the best attributes of both material classes.

### 3.3. Hybrid Compounds

Hybrid printable piezoelectric materials have emerged from a simple but powerful motivation: polymer-based inks provide mechanical flexibility, low-temperature processability, and compatibility with additive manufacturing, whereas ceramic, metallic, and carbonaceous fillers can enhance electromechanical coupling, dielectric response, β-phase formation, and stress transfer. By combining these features, hybrid systems can retain the softness and printability of PVDF- and P(VDF-TrFE)-based materials while partially approaching the electromechanical performance of higher-coupling piezoceramics such as PZT, BaTiO_3_, and lead-free ferroelectric oxides. Recent work has therefore expanded beyond simple two-phase mixtures toward interfacially engineered ceramic–polymer composites, metal and magnetic particle-modified PVDF, graphene/rGO/g-C_3_N_4_/CNT-containing systems, and porous laser-induced graphene electrode–polymer architectures. Across these systems, the dominant design variables are filler loading, particle or nanowire morphology, dispersion quality, interfacial adhesion, dielectric constant, β-phase fraction, and the ability of the printing or poling process to align dipoles and transfer mechanical stress efficiently.

PVDF–PZT and P(VDF-TrFE)–PZT composites remain among the most extensively studied hybrid systems for flexible piezoelectric materials because PZT provides high intrinsic piezoelectric activity, while the polymer phase maintains mechanical compliance and processability. Chen et al. [78] demonstrated this principle using blade-coated PZT/P(VDF-TrFE) hybrid films. After drying at 80 °C, annealing at 140 °C, and electrical poling, neat P(VDF-TrFE) showed a d_33_ of approximately 19.8 pC/N after poling, while the addition of 10 wt% PZT increased d_33_ to 22.1 pC/N. More importantly, surface modification of PZT using tetradecylphosphonic acid improved particle–polymer compatibility and raised d_33_ to 27.1 pC/N at 10 wt% modified PZT. This study shows that PZT loading alone is not sufficient; interfacial chemistry is equally important for converting ceramic piezoelectric activity into a useful composite response.

A related extrusion-cast PVDF/PZT system was reported by Zhang et al. [77], who compared neat PVDF, PVDF/PZT, surface-treated PVDF/PZT, PVDF/PZT/BNNS, and PVDF/PZT@105/BNNS films. In that work, neat PVDF exhibited d_33_ ≈ 4 pC/N, PVDF/PZT increased to approximately 9 pC/N, titanate coupling agent-treated PZT increased the response to approximately 15 pC/N, and the combined use of treated PZT with boron nitride nanosheets produced d_33_ ≈ 21 pC/N. This illustrates a second route to hybrid enhancement: ceramic particles improve the electromechanical response, coupling agents improve charge and stress transfer at the interface, and secondary fillers can improve crystallization, dielectric strength, and thermal management during processing or poling.

BaTiO_3_-based systems provide a lead-free alternative that is especially attractive for printed flexible devices. Zhou et al. [79] developed an all-3D-printed stretchable piezoelectric nanogenerator using BaTiO_3_ nanoparticles dispersed in P(VDF-TrFE). The printed BaTiO_3_/P(VDF-TrFE) layer was annealed at 120 °C and poled at 100 °C under 50 V/µm. A 20 wt% BaTiO_3_ formulation achieved d_33_ ≈ 20 pC/N, compared with approximately 16 pC/N for printed P(VDF-TrFE) without BaTiO_3_. Although the magnitude of the improvement is smaller than in some PZT-containing systems, the study is important because the material was integrated into an all-printed stretchable architecture, demonstrating the relevance of ceramic–polymer inks for digitally fabricated wearable piezoelectric devices.

Further improvements have been obtained by controlling ceramic morphology and alignment. Yan et al. [80] used direct ink writing to print P(VDF-TrFE) composites containing aligned lead-free BCZT nanowires. The printed films were dried at 70 °C for 24 h and poled at 60 °C under 10 kV/mm. At 15 wt% BCZT nanowires, the composite achieved d_33_ ≈ 30 pC/N, which was attributed to the combination of ceramic piezoelectricity, shear-induced nanowire alignment during printing, and improved stress transfer. Similarly, Li and Lim [81] screen-printed surface-modified BaTiO_3_/PVDF nanocomposites using triethoxy(octyl)silane-treated BaTiO_3_. After drying at 80 °C and poling at 110 °C under 50 V/µm, the optimized 20 wt% modified BaTiO_3_/PVDF composite reached d_33_ ≈ 33.5 pC/N, compared with 16.7 pC/N for printed PVDF. These results demonstrate that lead-free ceramic fillers can be highly effective when dispersion and particle–polymer adhesion are controlled.

High-solid-content printing has also become an important direction. Duan et al. [82] demonstrated direct ink writing of PVDF/BaTiO_3_ composites with BaTiO_3_ loadings as high as 70 wt%. The printed parts were thermally treated at 180 °C for 1 h. Although this study did not report d_33_, the optimized 70 wt% BaTiO_3_ composite produced an open-circuit voltage of 38.6 V and a short-circuit current of 750 nA, showing that high ceramic loading can substantially increase nanogenerator output when rheology and printability are maintained. In a complementary solvent-free approach, Kompelli et al. [83] fabricated PVDF-HFP/BaTiO_3_ filaments and printed devices by fused filament fabrication. The printing process used a nozzle temperature of 260 °C and a bed temperature of 70 °C, followed by thermal poling between 25 and 80 °C. Stable d_33_ values increased with BaTiO_3_ loading, reaching approximately 7.3 pC/N for 60 wt% BaTiO_3_ after optimized thermal poling. Although the d_33_ values were lower than those of solution-processed or surface-engineered composites, the study is notable because it demonstrates a scalable, solvent-free route for printed piezoelectric composites.

Metallic, magnetic, and metal-phosphate fillers have also been introduced into PVDF to promote β-phase formation, increase dielectric permittivity, and improve ferroelectric polarization through local electric-field effects. Bahloul et al. [84] reported PVDF nanocomposites containing lanthanated cobalt ferrite nanoparticles modified with polyvinylpyrrolidone. The composite films were solution cast from DMF and dried at 120 °C for 2 h. Although d_33_ was not reported, the optimized formulation achieved β-phase contents up to approximately 80%, together with improved mechanical and thermal properties. This type of magnetic particle modification is relevant because magnetic or ferrite-based fillers can act as nucleation sites for polar PVDF phases while also modifying dielectric behavior.

In a related study, Bahloul et al. [85] investigated PVDF composites filled with Ni-, Ag-, and Co-phosphate-based particles. The PVDF composites were doctor-bladed from DMF and dried at 70 °C for 2 h. Again, d_33_ was not reported directly, but the β-phase content increased strongly relative to neat PVDF, reaching up to approximately 96% for Ag-phosphate-filled PVDF at optimized loading. The same study also reported improved remnant polarization and mechanical reinforcement for selected metal-phosphate fillers. These results support the use of metal-containing fillers as β-phase nucleating agents, although future work should report d_33_ under standardized poling and measurement conditions to enable direct comparison with ceramic–polymer systems.

Carbon-based fillers provide a second major route for hybrid PVDF enhancement. Graphene, reduced graphene oxide, graphitic carbon nitride, and carbon nanotubes can act as β-phase nucleating agents because of their high surface area and strong interfacial interaction with PVDF chains. They can also improve mechanical reinforcement and dielectric response, although excessive loading may increase leakage current or disrupt dipole alignment. Wang et al. [86] demonstrated electric field-assisted direct ink writing of PVDF/graphene films. Printing was performed on a 70 °C heated bed while applying a 6 kV auxiliary electric field. The addition of only 0.05 wt% graphene increased the β-phase content to approximately 67.3% and produced a peak-to-peak voltage output of approximately 2.5 V, about five times that of pristine PVDF. The study did not report d_33_, but it clearly showed that low graphene contents can substantially enhance β-phase formation and device output in printed PVDF films.

Huang et al. [87] reported solution-processed PVDF/rGO ferroelectric composite films prepared through the in situ reduction of graphene oxide with hydrobromic acid. The films were dried at 70 °C and reduced at 90 °C. At 0.1 wt% rGO, the β-phase fraction approached 100%, the dielectric constant increased from approximately 10 to 41, and d_33_ increased from 22 pC/N for neat PVDF to 39.3 pC/N for the optimized PVDF/rGO composite. This result highlights the strong nucleating effect of rGO at very low filler content. Zhang et al. [88] used solid-state shear milling to disperse graphene in PVDF before conventional melt processing. Extrusion was performed at approximately 170–185 °C, followed by injection molding at approximately 180–190 °C. Although d_33_ was not reported, the approach increased the β-phase fraction after melt processing and produced very large dielectric enhancement, demonstrating that graphene dispersion methods can be adapted to scalable polymer processing routes.

More recently, printable carbon nitride and CNT-containing PVDF systems have shown promising d_33_ values. Pawar et al. [89] used direct ink writing to print ethanol-exfoliated g-C_3_N_4_/PVDF composites without external poling. The g-C_3_N_4_ was synthesized at 550 °C, while the PVDF composite ink was prepared at 60 °C. The optimized 7.5 wt% g-C_3_N_4_/PVDF film achieved d_33_ ≈ 15.9 pC/N and generated approximately 6–7 V under mechanical loading. Hou et al. [90] printed PVDF composites containing ionic liquid-functionalized carbon nanotubes. The printed specimens were thermally treated at 180 °C for 1 h. The optimized formulation achieved d_33_ ≈ 30 pC/N, an open-circuit voltage of 16.8 V, and a short-circuit current of 258 nA. These studies show that carbon-based hybrid fillers are no longer limited to β-phase nucleation studies; they are increasingly being integrated into printable, self-powered piezoelectric devices with measurable d_33_ performance.

A particularly relevant development for printed ultrasound transducers is the integration of P(VDF-TrFE) with laser-induced graphene. In these systems, the graphene is not only a conductive electrode but also a porous, mechanically compliant interfacial layer that can improve adhesion, stress transfer, local field distribution, and charge collection. This is especially important for flexible ultrasound transducers, where electrode delamination, acoustic impedance mismatch, and bending durability can limit device performance. Movaghgharnezhad et al. [91] fabricated flexible ultrasound transducers by combining CO_2_ laser-induced porous graphene on polyimide with printed P(VDF-TrFE). The P(VDF-TrFE) layer was dried and annealed at 140 °C for 20 min and corona-poled at 80 °C under fields up to 170 V/µm. The optimized films achieved d_33_ ≈ 23 pC/N and generated pulse–echo voltages up to approximately 6.7 V, with a signal-to-noise ratio of approximately 433. The porous LIG structure allowed partial infiltration of the polymer, increasing the effective interfacial area and reducing delamination under bending.

Hagen et al. [92] further examined the effect of electrode morphology by comparing Ag, Au, graphene-flake, LIG, and Au-decorated LIG electrodes under similar P(VDF-TrFE) processing conditions. The P(VDF-TrFE) was annealed at 140 °C for 20 min and corona-poled at 80 °C under 200 V/µm. LIG- and LIG/Au-based devices achieved d_33_ values of approximately 23–25 pC/N, maintained stable performance after 10,000 bending cycles, and produced strong acoustic outputs. These findings show that, for printed ultrasound transducers, hybridization should be understood not only as filler addition to the polymer matrix, but also as microstructural engineering of the electrode–piezopolymer interface.

Overall, hybrid printable piezoelectric materials have progressed from simple ceramic-filled PVDF films to interfacially engineered, microstructured, and fully printable composite systems. PZT and BaTiO_3_ fillers remain effective for increasing d_33_ when dispersion and adhesion are optimized; metal and phosphate particles provide strong β-phase nucleation and dielectric modification; graphene, rGO, g-C_3_N_4_, and CNTs combine β-phase enhancement with mechanical and electrical reinforcement; and LIG-based architectures directly address the electrode–polymer interface in flexible ultrasound transducers. The most successful systems are therefore those that simultaneously optimize filler functionality, polymer crystallization, interfacial coupling, printability, and post-processing conditions such as annealing and poling temperature.

### 3.4. Performance–Processing Trade-Offs in Printable Piezoelectric Materials

**Figure 3:**
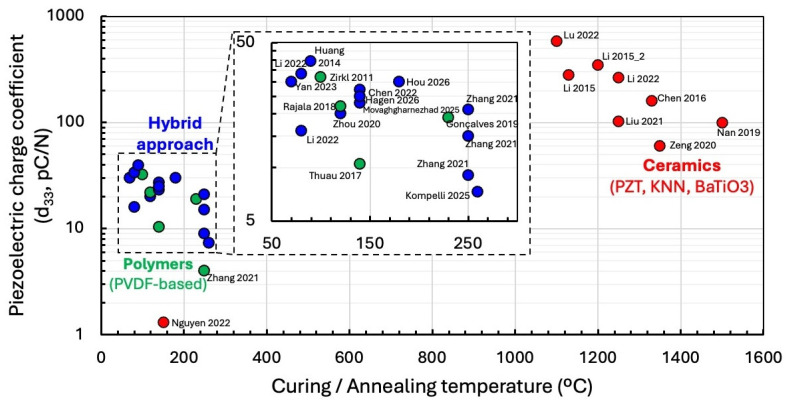
Scatter plot comparing curing temperature and transduction factor in works that propose printable piezoelectric compounds based on ceramics (in red), polymers (in green) and hybrid mixtures (in blue). Represented works include Liu 2021 [61], Li 2022 [62], Lu 2022 [63], Li 2015_2 [64], Nan 2019 [66], Li 2015 [67], Chen 2016 [68], Zeng 2020 [69], Nguyen 2022 [70], Zirkl 2011 [73], Thuau 2017 [74], Rajala 2018 [75], Gonçalves 2019 [76], Zhang 2021 [77], Chen 2022 [78], Zhou 2020 [79], Yan 2023 [80], Li 2022 [81], Kompelli 2025 [83], Huang 2014 [87], Hou 2026 [90], Movaghgharnezhad 2025 [91], Hagen 2026 [92].

The comparison shown in Figure 3 highlights a central theme that runs through all printable piezoelectric materials: the relationship between processing temperature and achievable piezoelectric performance is highly material dependent, and this dependency effectively divides the field into three distinct regimes—ceramic, polymer, and hybrid systems.

Ceramic-based printable compounds cluster in the upper right region of the plot, where high piezoelectric coefficients coincide with high thermal treatment temperatures. These materials routinely achieve d_33_ values above 60 pC/N, and in several cases far higher, but only after sintering at temperatures exceeding 1100 °C. The importance of this thermal step is underscored by the BaTiO_3_-based formulation [70] that omitted sintering and consequently produced a d_33_ of just 1.31 pC/N, demonstrating that without crystallization and densification, ceramic powders cannot express their intrinsic electromechanical potential. An emerging exception is the surface functionalization strategy, where chemically modified PZT particles can be printed and activated without high-temperature sintering while still achieving performance comparable to thermally processed ceramics. Although still in its early stages, this approach suggests a promising route toward low-temperature, high-performance ceramic architectures.

These general trends are reflected in specific case-to-case comparisons, where nominally similar processing temperatures can still yield markedly different electromechanical responses. A notable example of cross-study variability is the difference between the PZT-based DIW system reported by Liu et al. [61], which achieved a d_33_ of 103 pC/N, and the BaTiO_3_-based stereolithography system reported by Zeng et al. [69], which reached 60 pC/N despite comparable sintering temperatures. This discrepancy reflects differences in densification pathways, ceramic composition, and structural architecture rather than temperature alone. Liu et al. obtained a highly dense (97.8% theoretical) PZT structure with large, well-developed grains and epoxy infiltration that improved mechanical coupling. In contrast, the MIP-SL-printed BaTiO_3_ structures in Zeng et al. exhibited lower density, residual porosity from resin burnout, and a honeycomb geometry that reduces the effective ceramic volume fraction. These microstructural and architectural factors strongly influence electromechanical coupling and explain why similar thermal budgets can yield markedly different d_33_ values. This comparison illustrates why performance metrics reported in the literature must be interpreted within the context of microstructure, densification route, and architecture, rather than processing temperature alone.

Polymer-based printable materials occupy the lower left region of the plot. Their d_33_ values rarely exceed 30 pC/N, yet they require only modest thermal exposure—typically below 250 °C—to remove solvents and promote β-phase crystallization. This low-temperature compatibility enables integration with flexible substrates, textiles, and multilayer printed electronics, making polymers attractive for wearable and conformable devices even when their electromechanical performance is limited.

Hybrid materials fall between these two extremes. By embedding ceramic particles within a PVDF-based matrix, these composites achieve higher d_33_ values than polymer-only systems while maintaining low-temperature processing and mechanical flexibility. Their performance depends strongly on particle dispersion, interfacial bonding, and the ability of the polymer matrix to withstand high poling fields. Although hybrids do not yet match the performance of fully sintered ceramics, they offer a compelling compromise for applications requiring both flexibility and moderate piezoelectric sensitivity.

Taken together, the distribution of data points in the plot illustrates the fundamental trade-off that governs the design of printable piezoelectric transducers. Ceramic systems deliver the highest performance but demand high-temperature processing; polymers offer unmatched processability and mechanical compliance but lower sensitivity; and hybrid systems attempt to bridge the gap by combining the strengths of both. As new strategies such as surface functionalization, low-temperature densification, and engineered polymer–ceramic interfaces continue to mature, they are likely to expand the design space and enable printed piezoelectric materials with properties tailored to specific application requirements.

Although Figure 3 is useful for visualizing the processing–performance space of printed piezoelectric materials, it should be interpreted as a map of reported literature values rather than as a fully normalized ranking. Direct comparison of d_33_ across studies remains challenging because the reported values are influenced not only by material composition and processing temperature, but also by measurement method, loading frequency, sample thickness, electrode configuration, active volume, substrate constraint, and poling history. For example, d_33_ values measured using quasi-static or Berlincourt-type methods are not directly equivalent to device-level voltage outputs measured under dynamic loading, nor are they directly comparable to acoustic performance metrics such as the pulse–echo voltage, bandwidth, or signal-to-noise ratio. This distinction is particularly important for printed transducers, where apparent performance can be strongly affected by geometry, porosity, bending modes, interfacial stress transfer, and the mechanical boundary conditions imposed by the substrate.

Poling conditions also complicate the comparison. In Table 1, all reported poling fields were converted to kV/mm to improve consistency; however, this unit conversion should not be interpreted as a full normalization of piezoelectric performance. The relationship between poling field and d_33_ is not generally linear, because dipole alignment, domain switching, dielectric breakdown, coercive field, crystallinity, porosity, filler loading, and poling temperature all influence the final remanent polarization. Therefore, metrics such as d_33_ divided by poling field would be physically misleading unless complete poling curves and identical testing conditions were available. For this reason, the comparison in Figure 3 is used to identify broad material regimes and processing trade-offs rather than to establish absolute performance rankings.

This limitation highlights an important need for standardized testing and reporting in printable piezoelectric materials. Future studies should report not only d_33_, but also the measurement method, measurement frequency or force conditions, sample thickness, electrode area, device geometry, substrate constraint, poling field, poling temperature, poling duration, thermal treatment history, and, where relevant, density, porosity, β-phase fraction, filler loading, and filler morphology. Without these parameters, quantitative comparison across ceramic, polymer, and hybrid printed piezoelectric systems remains incomplete.

### 3.5. Need for Standardized Testing and Reporting

A major limitation in the current literature on printed piezoelectric materials is the lack of standardized testing and reporting. While d_33_ is widely used as a convenient figure of merit, it is not always measured under comparable conditions. Some studies report quasi-static or Berlincourt-type d_33_ values, whereas others report device-level voltage, current, power density, acoustic pressure, pulse–echo voltage, or signal-to-noise ratio. These metrics probe different aspects of the material–device system and cannot be directly interchanged. A high open-circuit voltage, for example, may reflect a large film thickness, low capacitance, or favorable loading geometry rather than a higher intrinsic piezoelectric coefficient.

Measurement frequency is another important but inconsistently reported parameter. Piezoelectric response can depend on loading frequency, especially in polymeric and hybrid systems where viscoelasticity, interfacial polarization, leakage, and mechanical relaxation can influence the measured signal. In ultrasonic transducers, the relevant operating frequencies are often in the MHz range, whereas d_33_ measurements are commonly performed at much lower frequencies. A material that performs well under quasi-static loading may therefore not necessarily produce equivalent performance in high-frequency acoustic operation.

Thickness and geometry also strongly affect apparent piezoelectric performance. Thin polymer films, thick printed ceramic lattices, dense monolithic structures, porous architectures, and interdigitated electrode devices experience different stress distributions and electrical boundary conditions. In thin films on rigid or flexible substrates, substrate clamping can reduce the effective piezoelectric response, while in cantilever-type devices bending-mode contributions can dominate the measured signal. Similarly, porous or lattice-like ceramic structures may reduce acoustic impedance and improve flexibility, but their effective d_33_ may differ substantially from that of a dense bulk ceramic with the same composition. These effects are particularly relevant for printed systems because geometry is often part of the functional design.

Poling conditions must also be reported consistently. Although the present review standardizes the poling field in Table 1 to kV/mm, the field magnitude alone does not fully define the poling process. Poling temperature, duration, electrode configuration, sample thickness, dielectric strength, and the surrounding medium all influence domain alignment or dipole orientation. In PVDF-based systems, poling temperature can increase chain mobility and improve β-phase dipole alignment, whereas in ceramics the relationship between the electric field and remanent polarization depends on the coercive field, grain size, porosity, and domain-wall mobility. Therefore, standardized field units improve readability, but do not fully normalize material performance.

For future work, printed piezoelectric materials should be reported using a minimum set of experimental descriptors that connect processing, structure, and performance. At minimum, studies should provide the measurement method used to determine d_33_, the measurement frequency or loading conditions, film or structure thickness, electrode area and geometry, substrate type, poling field, poling temperature, poling duration, thermal treatment conditions, and active material composition. For ceramic systems, density and porosity should also be reported; for PVDF-based systems, β-phase fraction and crystallinity are essential; and, for hybrid systems, filler loading, filler morphology, dispersion quality, and interfacial modification should be included. Such reporting would allow more meaningful comparison across studies and would help the field move from empirical material demonstrations toward predictive design rules for printed piezoelectric transducers.

## 4. Printing Techniques

Additive manufacturing has transformed how functional materials are shaped, patterned, and integrated into devices. Unlike conventional subtractive or mold-based fabrication, printing enables rapid iteration, low-waste processing, and the creation of geometries that would be difficult or impossible to achieve through traditional methods. These advantages have made printing particularly attractive for piezoelectric transducers, where device performance often depends on precise control of geometry, thickness, and material distribution. Several printing techniques have been adapted to piezoelectric inks, each offering distinct capabilities in terms of resolution, material compatibility, and achievable complexity.

Printable piezoelectric materials require different printing strategies depending on their composition, rheology, and processing constraints. Ceramic-based inks—typically high-viscosity slurries with substantial solid loading—are most compatible with extrusion-based methods such as direct ink writing (DIW) or with stereolithography when dispersed in photocurable resins. Polymeric systems such as PVDF and P(VDF-TrFE) are well suited to screen printing, stencil printing, inkjet printing, and film casting approaches due to their lower viscosity and solvent-based formulations. Hybrid ceramic–polymer composites occupy an intermediate regime: depending on filler fraction and ink rheology, they can be processed through DIW, screen printing, or inkjet printing. This mapping between material class and printing technique provides a useful framework for understanding the technique-specific examples discussed in the following subsections.

Patterning capability is a central advantage of printing technologies for piezoelectric devices, since many applications—such as sensor arrays, interdigitated electrodes, ultrasonic elements, and conformable wearable patches—require spatially defined active regions with controlled geometry and thickness. Printing enables the direct deposition of piezoelectric material only where it is functionally needed, eliminating the need for subtractive steps such as dicing or etching. Different techniques offer distinct patterning resolutions: inkjet printing can define features down to tens of micrometers, screen and stencil printing provide mask-defined patterns at the sub-millimeter scale, and direct ink writing allows freeform extrusion of filaments with programmable line width and height. These capabilities allow the fabrication of complex, application-specific layouts that are difficult to achieve using conventional bulk processing.

### 4.1. Direct Ink Writing

**Figure 4:**
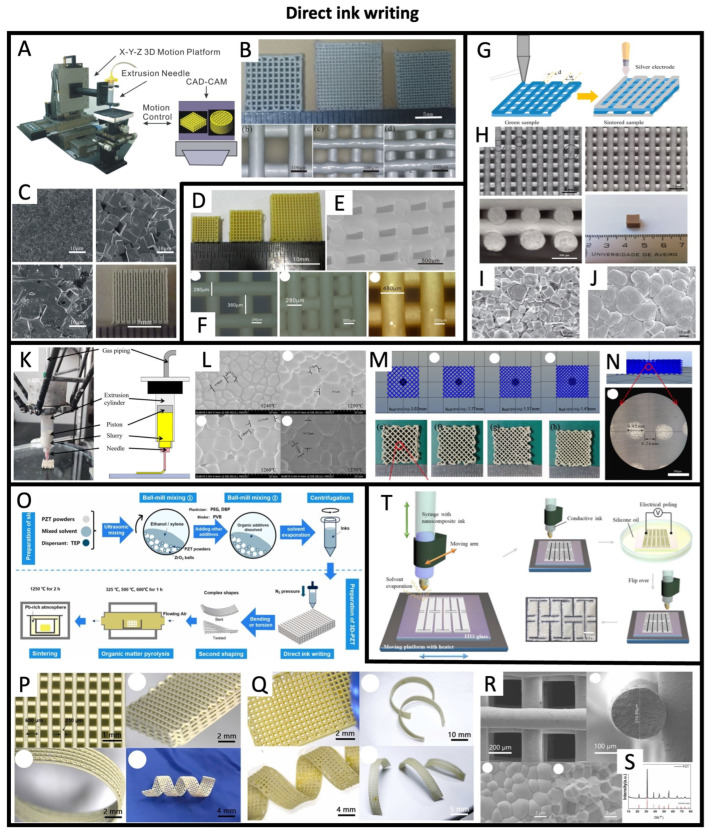
(**A**): Experimental set-up for the direct write printing process. (**B**): Optical images of the woodpile structures: top view of three samples with different sizes—10 mm sq, diam 400 μm; 12 mm sq, 250 μm diam; 10 mm sq, 300 μm diam. (**C**): SEM photos of KNN samples sintered at 1050 °C; 1100 °C; 1130 °C. And top view of the sintered 10 mm sq sample [67]. (**D**): Top view of sintered samples: 8 mm, 10 mm, and 15 mm. (**E**): Section of 8 mm structure. (**F**): Image of 8 mm sq sample, 280 μm diam; 10 mm sq, 280 μm diam; and 15 mm sq, 480 μm diam [64]. (**G**): Schematic of DIW sample. (**H**): Green BCZT filaments printed from pastes with different solid loadings: 40.0 vol%; 41.6 vol%; cross-section of green sample with 41.6 vol% solid loading; sample with 41.6 vol% solid loading and sintered at 1350 °C for 2 h. The sintered surfaces of samples sintered at (**I**): 1350 °C; and (**J**): 1500 °C [66]. (**K**): Direct ink writing of device and sketch map. (**L**): Cross-section morphology and grain size of samples sintered at 1240 °C, 1250 °C, 1260 °C and 1270 °C. (**M**): Models and sintered bodies with 20%, 23%, 26%, and 29% filling density. (**N**): Model cross section and size micrograph [61]. (**O**): Diagram of DIW of 3D PZT combined with secondary shaping of the flexible green body. (**P**): Photos of ceramic green bodies: woodpile structure, green body bent into annulus, and twisted into ribbon spiral. (**Q**): Optical photos of sintered 3D-PZT parts: woodpile structure scaffold, bent into annulus, or twisted into spiral, and unsupported structures with different spans. (**R**): Surface and section SEM image of sintered sample; (**S**): XRD pattern of the sintered sample [62]. (**T**): Schematic of the fabrication process of the all-3D-printed PENG [79].

A method that has gained popularity in recent years is direct ink writing (DIW). This technique uses a syringe or nozzle to deposit a viscous ink or paste onto a substrate in a controlled manner, layer by layer, to build complex structures. Li et al. 2015, [67] used an extrusion printer (shown in Figure 4A) to DIW multilayer ceramic transducers with trace widths as low as 250 μm (Figure 4B). Figure 4C shows how the micromorphology and macroscopic coloration of the KNN-printed transducers change with thermal sintering as crystal growth is induced. In another work (Li et al. 2015, [64]), the same authors used a similar approach to print a PLZT-based paste and were able to achieve trace widths as low as 280 μm, as shown in Figure 4D–F. Nan et al. 2019, [66] also used DIW to print a BCZT-based paste, as shown in Figure 4G–J. In this case, the printing was done on an aluminum oxide substrate immersed in low-viscosity glycerin oil, which ensured the homogeneous drying rate of the sample. Liu et al. 2021, [61] also showed DIW of a PZT-based slurry, achieving ~420 μm resolution, as depicted in Figure 4K–N. Li et al. 2022, [62] also used DIW and showed the printing and shaping of piezoelectric ceramics with intricate morphologies, as shown in Figure 4O–S. In this work, trace widths as low as ~210 μm were achieved. In Zhou et al. 2020, [79], DIW was used to print a (P(VDF-TrFE))-BaTiO_3_ polymer–ceramic hybrid mixture, as shown in Figure 4T.

### 4.2. Stereolithography and Projection Micro-Stereolithography

**Figure 5:**
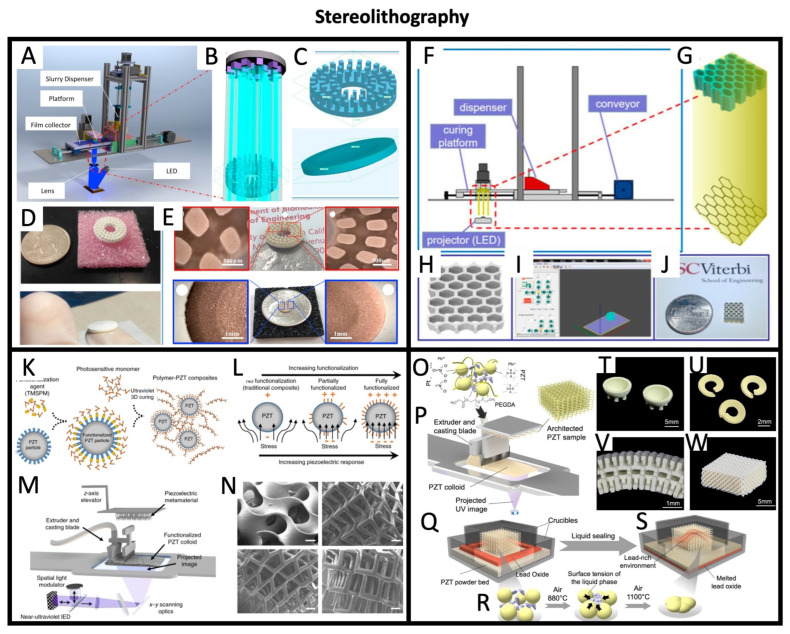
(**A**): MIP-SL system. (**B**): Imaging pattern controlled by the projection. (**C**): 3D design SolidWork. (**D**): Images of green part fabricated using MIP-SL. (**E**): Microscopy images of 3D-SAA with 64-pillar annular segment array (**top**) and concaved-shaped piezoelectric element (PF-CPE) (**bottom**) [68]. (**F**): MIP-SL system to print green parts. (**G**): Sliced 2D pattern of a 3D model projected by a digital light projector. (**H**): CAD model of the printed sample with honeycomb structure. (**I**): GUI of the MIP-SL system developed in-house. (**J**): Green part fabricated using MIP-SL [69]. (**K**): Illustration of surface functionalization method and strong bonds between the nanoparticles and the polymer matrix after the ultraviolet curing process. (**L**): Illustration of the relationship between the surface functionalization level and the piezoelectric response. The piezoelectric response increases with the surface functionalization level as a result of increasing stress transfer. (**M**): Illustration of the high-resolution additive manufacturing system. (**N**): SEM images of 3D-printed piezoelectric microlattices. Scale bars, 300 µm [71]. (**O**): Schematic of the liquid sintering resin components. (**P**): Novel 3D printing system for liquid phase sintering piezoelectric composites. (**Q**): Debonding process to burn off the supportive polymer. (**R**): Liquid sealing process to reduce the lead loss during high-temperature sintering. (**S**): LPS process to form dense PZT sample. PZT free-form fabrication of (**T**): hemisphere elements used in a general ultrasound transducer for medical imaging and nondestructive testing; (**U**): cylindrical transducer element with multiple concentric annular layers; (**V**): lattice sensor element for low-frequency (<100 kHz) ultrasound; and (**W**): force sensor element with ultrahigh sensitivity [63].

Stereolithography is a printing method that has gained attention in recent years for its ability to produce high-resolution, complex structures. In the case of masked image projection stereolithography (MIP-SL), this technique uses a digital mask to selectively illuminate a layer of photosensitive resin, which then solidifies in the desired pattern. The process is repeated layer by layer until the complete structure is formed. To create piezoelectric transducers through stereolithography, a ceramic powder is mixed with the photocurable resin that will be hardened in the desired shape through the stereolithography process. In most cases, the resin is then burnt off before sintering the ceramic to enable crystal growth, as shown for instance in Chen et al. 2016, [68]. In this work, which uses MIP-SL, as shown in Figure 5A–E, an X-Y resolution of 20 μm and *Z*-axis resolution of 10 μm are reported, which allow for very intricate designs of transducers. Later, in Zeng et al. 2020, [69] (Figure 5F–J), from the same group, a similar printing setup was described, allowing for the fabrication of honeycomb-shaped transducers that showed reduced acoustic impedance compared to bulk structures fabricated with traditional techniques.

Similarly to MIP-SL, high-resolution projection micro-stereolithography (PμSL) systems have also been used in the printing of piezoelectric ceramic-based transducers. In this case, instead of creating a digital mask on the liquid resin surface through a projector, a micromirror is used to guide a focused UV beam through a predetermined path on the resin’s surface, allowing for very intricate designs and a high resolution, as shown in Cui et al. 2019, [71], in which the authors fabricate various very detailed lattice structures, shown in Figure 5K–N. The same PμSL method has also been used in Lu et al. 2022, [63] for the implementation of unconventional curved transducers, shown in Figure 5O–W.

### 4.3. Screen Printing and Related Techniques

**Figure 6:**
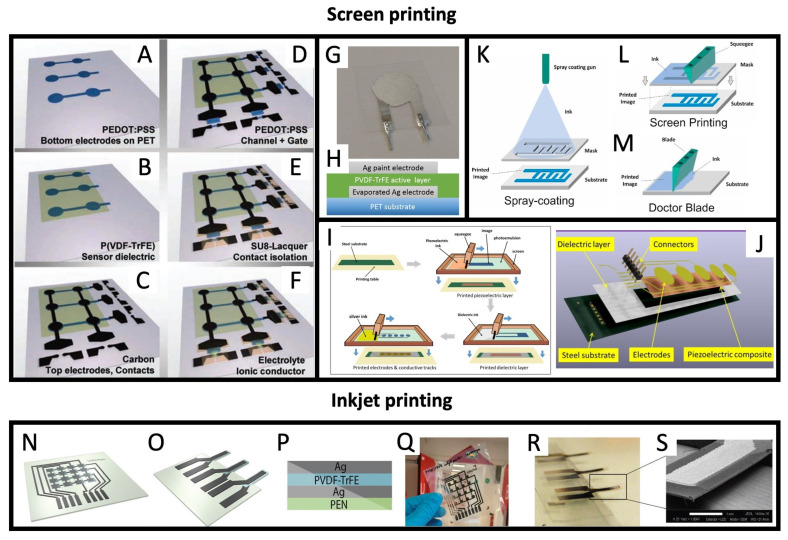
(**A**): Screen printing with PEDOT:PSS to form the bottom electrodes of the sensor pixel. (**B**): Screen printing of the ferroelectric P(VDF-TrFE) film. (**C**): Screen printing of carbon to form the top electrodes of the sensor pixel. (**D**): Inkjet printing with PEDOT:PSS to form the gate and channel of the ECTs. (**E**): Inkjet printing of the SU-8 separation layer. (**F**): Inkjet printing of the ECT polymeric electrolyte [73]. (**G**): Photograph and (**H**): schematic cross-sectional view of screen-printed P(VDF-TrFE) sensor. Note that the P(VDF-TrFE) layer and the PET substrate are transparent [75]. (**I**): Fabrication of multilayered piezoelectric sensor via screen printing process. (**J**): Architecture of piezoelectric sensor for condition monitoring of substrate [70]. (**K**): Schematic representation of the different techniques and procedures used for the preparation of piezoelectric films, which include (**K**,**L**): stencil printing and (**M**): doctor blading [76]. (**N**): Schematic of fabricated flexible inkjet-printed piezoelectric sensor, (**O**): actuator, (**P**): cross section of the printed devices, (**Q**): optical image of flexible pressure sensors, (**R**): optical image of the cantilever piezoelectric actuator, and (**S**): SEM image of a free-standing piezoelectric cantilever beam [74].

Screen printing is a well-established and versatile printing technique that involves transferring inks or pastes onto a surface through a stencil or mesh screen. Being an efficient, easily scalable and low-cost printing method, it has also been used to deposit piezoelectric slurries with high precision and accuracy, as shown in Zirkl et al. 2011, [73]. In this work, the authors describe a multi-step printing sequence (shown in Figure 6A–F) using five distinct functional inks which include a P(VDF-TrFE) ink, PEDOT:PSS, a conductive carbon paste, a polymeric electrolyte, and SU-8 photoresist. In this work, the thickness of the printed piezoelectric layer is reported as 5 μm. Rajala et al. 2018, [75] also reported on the use of stencil printing with a commercial P(VDF-TrFE) ink and Ag paint to achieve the transducers that are shown in Figure 6G,H. More recently, in Nguyen et al. 2022, [70], a fully screen-printed stack of piezoelectric BaTiO_3_-based slurry and conductive ink was reported, as depicted in Figure 6I,J. In Gonçalves et al. 2019, [76], the deposition of a P(VDF-TrFE) through a stencil was done both using spray coating and a conventional squeegee, as shown in Figure 6K,L. In addition, doctor blading was used to form piezoelectric films with uniform thickness, as shown in Figure 6M.

### 4.4. Inkjet Printing

Another method that has been reported in Thuau et al. 2017, [74] for the controlled deposition of piezoelectric transducers is inkjet printing. In this case, small droplets of ink are dispensed in a controlled manner to create the desired pattern on a surface. In this work, the authors explain the need to adapt conventional P(VDF-TrFE) pastes based on cyclopentanone solvent to the specificities of inkjet printing. Cyclopentanone, having a low density and high volatility, would rapidly clog the inkjet cartridge and, as such, it has been mixed with dimethyl sulfoxide (DMSO), which has a high boiling point and low vapor pressure. The inkjet-printed transducers are reproduced in Figure 6N–S.

### 4.5. Film Casting Methods

Although spin casting and extrusion casting are not additive manufacturing techniques in the strict sense, they are included here because they are widely used to fabricate the same polymer-based piezoelectric films that serve as inks or functional layers in printed transducers. These methods share key processing considerations with printing—such as solvent evaporation, crystallization behavior, film uniformity, and thickness control—and are frequently used in studies that compare cast films with printed films or use cast layers as benchmarks. For this reason, film-casting methods are discussed alongside printing techniques to provide a complete overview of the fabrication routes relevant to printable piezoelectric materials.

Although not being strictly considered printing techniques, spin casting and extrusion casting enable the creation of piezoelectric films from the aforementioned slurries and pastes. Spin casting consists of pouring a paste into a plate rotating at high speeds. The centrifugal force distributes the paste evenly on the plate’s surface with a thickness dependent on the rotation velocity. This method has been shown in Brown et al. 1997, [72], where P(VDF-TrFE) film thicknesses of 6–25 μm are reported. Extrusion casting consists of expelling a molten material through a circular die, creating a tubular bubble of plastic material. The bubble is then pulled vertically and simultaneously inflated, allowing it to stretch and thin out into a flat sheet of plastic film. In Zhang et al. 2021, [77], this technique is employed to form piezoelectric films based on PVDF, as it enables the production of large-area, good-density polymeric films.

### 4.6. Summary

Printing and additive manufacturing techniques are revolutionizing the production of customized and complex parts across multiple fields by offering several advantages, which include smaller production runs, rapid iterations, and the possibility of forming complex structures at relatively low cost, resulting in more sustainable production with less waste than traditional subtractive methods. In the fabrication of piezoelectric transducers, various printing techniques, such as direct ink writing, stereolithography, and screen printing, have been utilized. Direct ink writing is a popular method, enabling the controlled deposition of viscous ink or paste onto a substrate, while stereolithography and high-resolution projection micro-stereolithography offer valuable benefits related to a higher resolution of printing. Additionally, screen printing is an established and versatile technique that provides high precision and accuracy when depositing piezoelectric slurries. In summary, the advantages of printing and additive manufacturing techniques can benefit the field of piezoelectric transducers by reducing fabrication cost and increasing flexibility in the shape and design of more intricate transducers that would be difficult or impossible to create with traditional manufacturing techniques.

Compared to conventional fabrication routes such as tape-casting, mold-based polymer processing, and bulk ceramic machining, printing offers several clear advantages: mask-free patterning, rapid iteration, reduced material waste, and the ability to create application-specific geometries that are difficult to achieve through subtractive methods. These benefits make printing particularly attractive for flexible, conformable, or miniaturized transducers. However, printing also introduces limitations relative to conventional processing. Ceramic-based inks often require high-temperature sintering, printed films may exhibit higher porosity or lower crystallinity than bulk materials, and hybrid inks can suffer from particle agglomeration or interfacial dielectric breakdown. Thus, printing expands design freedom but can impose constraints on material performance, highlighting the need for the continued optimization of ink formulation and post-processing.

## 5. Applications

Printed piezoelectric transducers have begun to demonstrate their versatility across a wide range of emerging applications, from energy harvesting and actuation to mechanical sensing and ultrasonic/acoustic transduction. Because printing enables thin, lightweight, and geometrically complex structures, it allows piezoelectric materials to be deployed in contexts that were previously inaccessible to conventional bulk ceramics or laminated polymer films. The following sections synthesize the most representative demonstrations to date, highlighting how the characteristics of each printable piezoelectric compound influence its suitability for specific use cases.

### 5.1. Energy Harvesting

**Figure 7:**
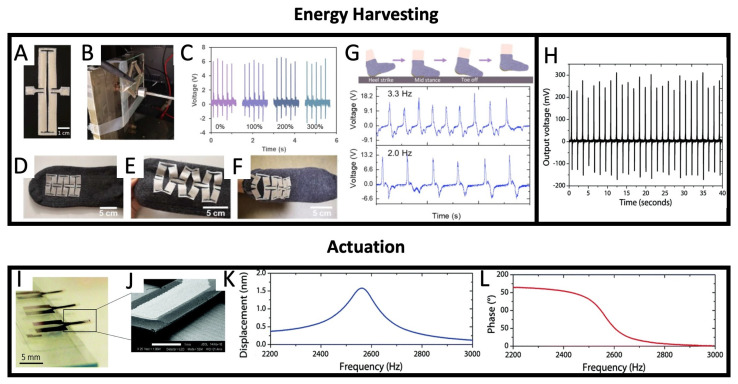
(**A**): Printed piezoelectric energy harvester with the structure of one repeating unit of T-joint-cut kirigami structure. (**B**): Printed piezoelectric energy harvester mounted onto the shaker for measurement of voltage output. (**C**): Voltage output of printed T-joint-cut structure sample with overall strain of 0%, 100%, 200% and 300%. (**D**): Optical image of the all-printed PENG mounted on a sock without stretching and (**E**): with stretching. (**F**): Photo of the all-printed PENG mounted on the heel of a sock wearing on a foot. (**G**): Schematic and voltage output of the printed PENG-mounted sock under foot stamping with different frequency [79]. (**H**): Open-circuit output voltage generated by the P(VDF-TrFE) thin film in response to finger touch pressure. (**I**): Optical image of the cantilever piezoelectric actuator. (**J**): SEM image of a free-standing piezoelectric cantilever beam. (**K**): Amplitude of displacement and (**L**): phase spectra of the first out-of-plane flexural mode of resonance of a piezoelectric cantilever resonator [74].

Printed piezoelectric transducers exhibit significant potential in the realm of energy harvesting, where they are capable of converting mechanical vibrations and motion into electrical energy. This can be harnessed to power miniaturized electronics, sensors, and IoT devices in remote or challenging-to-reach locations, thus decreasing the necessity for battery replacement and maintenance. Zhou et al. 2020, [79] propose an all-3D-printed stretchable piezoelectric nanogenerator (PENG), shown in Figure 7A–G, based on BaTiO_3_ NPs and a P(VDF-TrFE) matrix, and silver flake-based electrodes. By creating a T-joint-cut kirigami structure, the authors report that the PENG can be stretched to more than 300% strain without out-of-plane deformation, showing a great potential for application in wearable electronic systems. A self-powered gait sensor is demonstrated, and it is shown that the PENG can generate up to 6 V of open current voltage and a short circuit current of 2 μA/cm^2^. Moreover, the maximum reported power density was 1.4 μW/cm^2^ when the load resistance was 10^7^ Ω. Thuau et al. 2017, [74] reported on a 2 μm thick piezoelectric P(VDF-TrFE) film which, under the applied finger pressure estimated to be of a few kPa, generated an average output voltage of 250 mV, as shown by Figure 7H.

When viewed in the broader context of flexible piezoelectric energy harvesters, the performance of the printed systems reviewed here remains below the state of the art. Fully optimized PVDF-based nanogenerators routinely achieve power densities one to three orders of magnitude higher when enhanced through microstructural engineering. For example, biomass-derived carbon fillers incorporated into electrospun PVDF nanofibers have recently enabled triboelectric nanogenerators with power densities approaching 0.92 W m^−2^ [93], while sono-chemically exfoliated MoSe_2_–PVDF nanocomposites have demonstrated 680 µW cm^−2^ under moderate pressures [94]. Hybrid PVDF–PZT electrospun composites also show significantly higher performance, with optimized 33 vol% PZT nanogenerators reaching 0.97 µW cm^−2^ and delivering voltages more than five times higher than pristine PVDF [95]. Even inorganics-on-polymer systems outperform printed hybrids: compositionally graded flexible PZT thin films can deliver current densities of 10 µA cm^−2^ and voltages of ~2 V [96], while sputtered ZnO thin-film generators on PET substrates achieve 14 µW output power [97]. These benchmarks highlight that the comparatively low output of printed BaTiO_3_/PVDF systems arises from their reduced crystallinity, residual porosity, limited β-phase content, and weaker electromechanical coupling. Closing this performance gap will require improved densification strategies, enhanced polymer crystallization, and engineered ceramic–polymer interfaces tailored for efficient stress transfer.

### 5.2. Actuation

As actuators, piezoelectric transducers convert electrical energy into accurate mechanical displacement or motion, facilitating their use in micro- and nano-positioning systems, adaptive optics, and robotics. The use of printable compounds expedites the implementation of such actuators with compact dimensions, rapid response, and low power consumption, as shown in Thuau et al. 2017, [74]. In this work, the authors showed printed cantilever resonators with 7 mm length, 3 mm width and 5.2 μm thickness; see Figure 7I,J. The spectra of the first out-of-plane flexural mode of resonance measured at 2560 Hz for the fabricated cantilevers are illustrated in Figure 7K,L.

### 5.3. Mechanical Sensing

**Figure 8:**
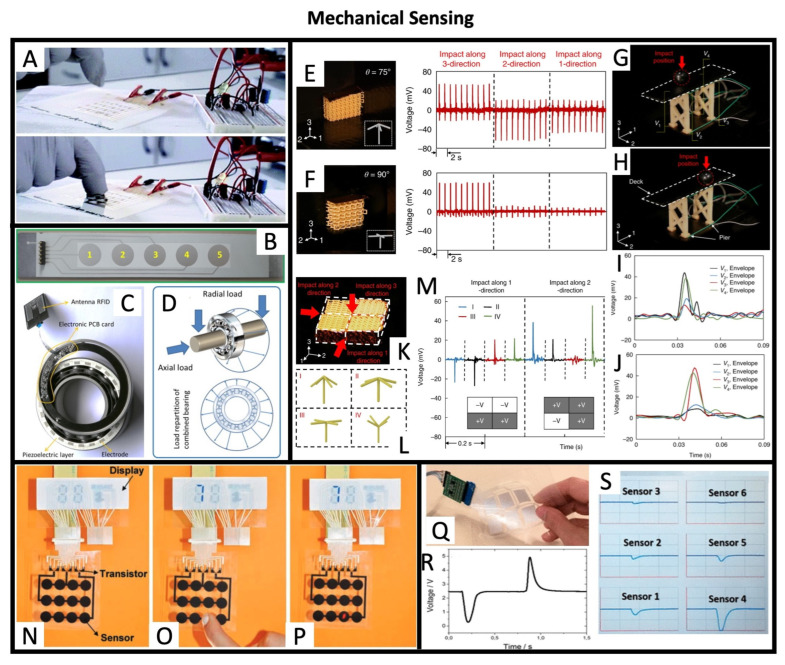
(**A**): Images depicting the capability of the inkjet piezoelectric film-generated voltage pulse to act as a switch activating the output of the NE555P timer to turn on a yellow LED by a single finger touch [74]. (**B**): Screen-printed piezoelectric sensor for condition monitoring of substrate. (**C**): Smart piezoelectric sensors integrated in ball bearing elements. (**D**): Schematic illustration of load repartition exerted on bearing [70]. (**E**,**F**): Images of representative piezoelectric metamaterials and their corresponding real-time voltage outputs under impact coming from the 1-, 2- and 3-directions. (**G**,**H**): Image of a self-monitoring 3D-printed piezoelectric bridge infrastructure. All four piers are poled together along the 3-direction, and the electrodes are attached to the top and bottom surface of the piers. V1 to V4 denote the voltage output between the corresponding electrodes. The locations of the dropping steel ball are indicated with dashed lines. (**I**,**J**): Real-time voltage outputs from the self-monitoring piezoelectric piers. (**K**): Piezoelectric infrastructure comprising (**L**): stacked architectures with encoded piezoelectric constants. (**M**): Voltage output patterns corresponding to different impact directions indicated by red arrows. The insets show the binary voltage patterns registered with different impact directions. The impact force in the 1-direction is registered with permutation voltage matrix [−,−,+,+], and with [+,+,−,+] for the 2-direction [71]. (**N**): Photograph of a printed active-matrix sensor consisting of a 3 × 4 array of sensor capacitors integrated with a row of electrochemical transistors connected to a printed electrochromic display. (**O**): Activation of display segments due to infrared radiation from a human finger. (**P**): Activation of display segments with light from a laser pointer [73]. (**Q**): All-printed piezoelectric touch screen comprising a 30 μm thick PVDF-TrFE film and six silver paint-printed electrodes. (**R**): Touch pad response upon finger pressing and release. (**S**): Sensor matrix on touch event in one of the sensors and corresponding signals for each sensor [76].

Mechanical sensing is another application area where printed piezoelectric transducers can play a vital role. In recent years, piezoelectric sensors have been integrated into various applications, such as pressure sensing, vibration detection, and the monitoring of structural health. In Thuau et al. 2017, [74], apart from the previously referenced demonstrations in the domains of actuation and energy harvesting, pressure sensing is also shown. In this work, a simple touch sensor is used to control an LED, as shown in Figure 8A. Nguyen et al. 2022, [70] reported on a screen-printed piezoelectric sensor for bearing load monitoring, as shown in Figure 8B–D. According to the authors, this novel approach presents a higher sensitivity and easier integration than classical piezoresistive technology since it can be directly integrated in existing bearing and does not require complex signal conditioning since the printed sensor provides a direct voltage signal. Moreover, the developed sensor presented high repeatability at various operational temperatures up to 120°C. Cui et al. 2019, [71] described the 3D printing of a metamaterial that can sense the magnitude and direction of applied forces (Figure 8E,F), while simultaneously absorbing impacts and monitoring its own mechanical state, allowing for self-diagnostic capabilities in various structural applications, as demonstrated by the bridge model in Figure 8G–J. Moreover, by combining various printed cells with different architectures, a three-dimensional pressure mapping sensor was implemented (Figure 8K–M), which could provide valuable pressure intensity and directionality data in applications such as robotics, wearable devices, and other systems that require detailed information about the mechanical environment they operate in. Zirkl et al. 2011, [73] demonstrated a fully printed smart active matrix sensor array which used P(VDF-TrFE) as the dielectric in capacitive sensors. Taking advantage of the pyro- and piezoelectric sensitivity of the polymer, a change in temperature or pressure in the transducer generates charges that give rise to a voltage response at the electrodes of the sensor capacitor, as shown in Figure 8N–P. Rajala et al. 2018, [75] also proposed pressure sensors that, despite the modes normal-mode sensitivity (<25 pC/N), showed remarkably high bending-mode sensitivities up to 200 nC/N that make it ideal for use in cantilever setups for low-force detection in single-fiber or living-cell-level measurements, as suggested by the authors. More recently, in Gonçalves et al. 2019, [76], the authors propose a multitouch detection 2 × 3 sensor matrix (Figure 8Q,R) that shows an almost noise- and hysteresis-free output signal when touched by a human finger, despite the occurrence of cross-talk in the sensors due to film vibration on the finger touch, as shown in Figure 8S for the case when sensor 4 was pressed.

### 5.4. Ultrasonic and Acoustic Transduction

**Figure 9:**
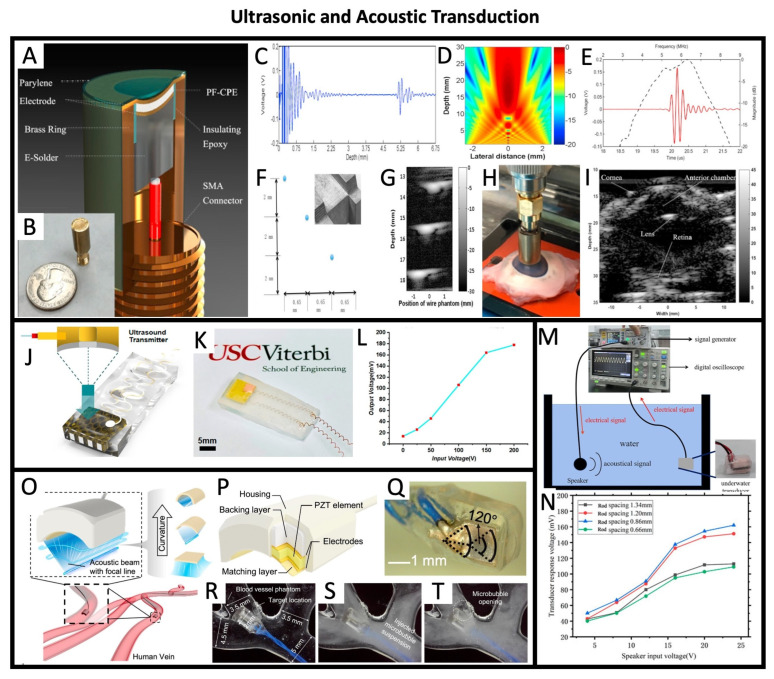
(**A**): Profile of the transducer structure. (**B**): Optical image of the printed ceramic-based transducer. (**C**): Initial pulse and echo generated by the printing-focused transducer. (**D**): Beam profile simulation by Field II program. (**E**): Pulse–echo waveform (solid line) and normalized spectrum. (**F**): Schematic of tungsten wire (50 um diameter) phantom. (**G**): Phantom imaging using the 6.28 MHz transducer. (**H**): The 6.28 MHz ultrasonic scan through porcine eyeball using the printing-focused transducer. (**I**): Ultrasonic imaging of porcine eyeball [68]. (**J**): Schematic and design of the ultrasonic device. (**K**): Optical image of the fabricated device. (**L**): Trend of the output voltage for different input voltages [69]. (**M**): Signal receiving test of underwater acoustic transducer. (**N**): Comparison of signal generator input and digital oscilloscope output [61]. (**O**): Schematic of the fabricated miniatured ultrasound transducer with curved PZT elements. (**P**): Schematic image of a 3D-printed miniaturized ultrasound transducer. (**Q**): Optical image of a 3D-printed miniaturized ultrasound transducer with a focused micro-PZT element. (**R**–**T**): Ultrasound-induced microbubble opening (cavitation) via the proposed 3D-printed miniaturized ultrasound transducer in a 3D-printed blood vessel phantom [63].

The capacity of piezoelectric transducers to generate or detect acoustic and ultrasonic waves has resulted in their use in a broad array of devices, including microphones, speakers, and ultrasonic sensors. Applications range from underwater communication and non-destructive testing to medical imaging, and authors have taken advantage of facile printing techniques to create transducers with complex shapes that can, for instance, lead to more focused ultrasound beams. Chen et al. 2016, [68] proposed a 3D-printed ceramic piezo element integrated into a full ultrasound transducer, as shown in Figure 9A,B. As described by the authors, the concave transducer morphology lends itself to a focused beam profile and a center frequency of 6.28 MHz (Figure 9C–E). In this work, the authors demonstrate the use of the fabricated transducer for imaging a tungsten wire phantom (Figure 9F,G) and also demonstrate its applicability in the field of biomedical imaging by scanning an ex vivo porcine eye (Figure 9H,I). In another work from the same lab (Zeng et al. 2020, [69]), the authors described the integration of a 3D-printed honeycomb-shaped piezo ceramic element into a full ultrasonic transducer, shown in Figure 9J,K. The proposed transducer presents promising material performance and output power properties for ultrasound sensing (i.e., output voltage reached 180 mVpp, as depicted in Figure 9L), along with the possibility of implementing piezoelectric composites with complex structures that cannot be fabricated by traditional methods of dicing–filling. The ceramic PZT-printed element described in Liu et al. 2021, [61] has been integrated into a full transducer using copper foil as the conductive electrodes and polyurethane as the matching layer due to its acoustic impedance being close to the one of water. To assess the acoustic wave-to-electrical signal conversion capability of the fabricated underwater acoustic transducer, a signal generator was used to produce electrical signals with varying waveforms and frequencies, which were fed into a speaker to emit corresponding sound signals. The transducer was positioned 450 mm away from the speaker, and the signal was displayed on a digital oscilloscope, as shown in Figure 9M. As shown in Figure 9N, after receiving the acoustic signal, the underwater acoustic transducer converts it into a response voltage (VPP) that, as expected, increases with the increase in the amplitude of the emitted acoustic signal. In Lu et al. 2022, [63], the authors proposed a fully printed miniaturized piezoelectric ultrasound transducer, including backing layer, impedance matching layer and physical housing, capable of acoustic focusing and localized cavitation within millimeter-sized channels, as shown in Figure 9O–T. This transducer was capable of generating high and localized acoustic pressure in blood vessels with diameters as low as 2 mm, allowing localized cavitation triggering, enhanced drug delivery, and the ultrasonic modulation of cellular activity. Beyond drug delivery, the authors also describe that the localized acoustic energy output could enable future applications, which include intravascular thrombolysis, in situ imaging, neuromodulation, sonogentic control, and oncology applications.

The fact that ceramic-based compounds present a higher transduction factor led to them being used more extensively for ultrasonic applications, as described previously, while polymeric-based piezoelectric transducers have been almost exclusively used for sensing. Nevertheless, Brown et al. 1997, [72] focused on the direct formation of high-performance PVF films onto substrate materials suitable for fabricating high-performance broadband ultrasound transducers without the need for adhesive layers. When moving away from printable compounds, only a couple of works have proposed PVDF-based ultrasonic transducers [98,99,100], as there are concerns with the limited penetration of the acoustic waves and higher voltages being needed to achieve similar acoustic power, as with ceramics.

Across the printed ultrasonic transducers reviewed here, the key performance figures of merit—center frequency, −6 dB bandwidth, and insertion loss—show a consistent pattern: printed devices can reach clinically relevant frequencies but still trail bulk-machined ceramics in bandwidth and energy efficiency. The MIP-SL BaTiO_3_ transducer of Chen et al. (2016) [68] operates at 6.28 MHz with a 41% bandwidth, values comparable to commercial unfocused BaTiO_3_ elements, though the printed device exhibits higher attenuation and therefore a modestly higher effective insertion loss than bulk ceramics. The honeycomb BaTiO_3_ composite of Zeng et al. (2020) [69] shifts toward lower-frequency sensing, with a 1.6 MHz resonance and an output of ~180 mVpp, but its bandwidth remains narrower than that of dense monolithic ceramics due to the composite’s reduced coupling. The DIW-printed PZT composites of Liu et al. (2021) [61] similarly show functional underwater transduction, with output voltage scaling cleanly with acoustic drive, yet their effective bandwidth and sensitivity remain limited by porosity and rod spacing-dependent defects. In contrast, the fully densified PZT micro-transducers of Lu et al. (2022) [63] approach bulk ceramic performance, achieving 9.75 MHz operation and acoustic pressures exceeding 1 MPa, though even here the −6 dB bandwidth is narrower than that of conventionally machined PZT due to residual microstructural heterogeneity. For comparison, commercial and state-of-the-art bulk PZT transducers [101,102,103,104] at similar frequencies routinely achieve 50–70% bandwidth and substantially lower insertion loss, reflecting the advantages of fully dense, crack-free ceramics with optimized domain structures. Together, these results highlight that, while printed transducers can now match bulk devices in center frequency, they still lag in bandwidth and insertion loss, metrics that remain tightly coupled to densification, grain connectivity, and defect control during printing and sintering.

The device-level performance of these printed transducers can be directly traced to the underlying material and microstructural characteristics. The piezoelectric charge coefficient d_33_ and dielectric permittivity ε_r_ jointly determine the electromechanical coupling factor k_t_, which governs the acoustic output pressure, received sensitivity, and overall transduction efficiency [105,106,107,108]. A high d_33_ increases charge generation, whereas a high ε_r_ increases capacitance and reduces voltage output; thus, printed ceramics with a high d_33_ but moderate ε_r_ often achieve a stronger transmit pressure than polymer-based devices. Processing-induced porosity and constrained grain growth—common in DIW and stereolithography—lower acoustic impedance and broaden bandwidth, but also reduce coupling and increase insertion loss due to scattering and reduced domain mobility [109,110,111,112,113]. In polymer-based films, the fraction of electroactive β-phase and the degree of crystallinity directly control sensitivity and the noise floor: a higher β-phase improves dipole alignment and voltage output, while low crystallinity increases dielectric loss and reduces the signal-to-noise ratio [114,115,116]. Hybrid composites introduce additional complexity: ceramic loading fraction, interfacial adhesion, and filler dispersion determine stress transfer efficiency, and poorly bonded interfaces or agglomerated fillers reduce effective d_33_, bandwidth, and output voltage [117,118,119]. These material–processing–performance relationships explain why printed transducers often achieve functional center frequencies but still lag behind bulk-machined ceramics in bandwidth, insertion loss, and acoustic output pressure.

While these demonstrations highlight the promise of printed piezoelectric transducers, their performance still lags behind that of conventionally machined ceramics in key metrics such as bandwidth, insertion loss, and acoustic output pressure. These limitations stem from residual porosity, incomplete densification, and geometric constraints imposed by printing resolution. Several studies report center frequencies comparable to bulk devices, yet the electromechanical coupling and noise floor remain strongly dependent on microstructural defects introduced during printing. Bridging this gap will require not only improved ink formulations but also a deeper understanding of how printing physics governs device-level electromechanical behavior.

## 6. Conclusions and Future Directions

Printable piezoelectric materials have expanded the design space of electromechanical transducers in ways that were not possible with traditional ceramic machining or polymer lamination. A central contribution of this review is the consolidation of printable piezoelectric materials into an ink-to-transducer framework that links formulation, processing, microstructure, and device-level behavior. By comparing ceramic, polymer, and hybrid systems under a common set of processing and performance considerations, the review highlights cross-cutting principles that are not captured in materials-only or printing-only surveys. This perspective clarifies how printability constraints shape achievable electromechanical performance and where future opportunities lie. By enabling digitally patterned, application-specific geometries, printing allows piezoelectric devices to be fabricated rapidly, at low cost, and in forms tailored to their intended mechanical, acoustic, or physiological environment. Across sensing, energy harvesting, actuation, and ultrasonic transduction, the works reviewed here demonstrate that printability is not merely a manufacturing convenience but a functional enabler: it allows piezoelectricity to be embedded into lattices, shells, kirigami structures, and conformable films that respond to stimuli in fundamentally new ways.

A recurring challenge in the field is that many reported improvements are highly system-specific and do not yet constitute generalizable design rules. For example, enhancements attributed to β-phase content, particle loading, or surface functionalization often depend strongly on the specific processing history and cannot be reliably extrapolated across material systems. The field would benefit from more systematic studies that isolate the effects of ink rheology, crystallinity, porosity, and interfacial chemistry on device-level performance. Without such frameworks, progress will continue to rely on empirical optimization rather than predictive materials engineering.

Despite this progress, the field remains shaped by a set of persistent trade-offs. Ceramic-based printable compounds continue to deliver the highest piezoelectric coefficients, yet their reliance on high-temperature sintering restricts substrate choice and complicates integration with flexible or multilayer printed electronics. Polymer-based systems offer mechanical compliance, low-temperature processing, and compatibility with large area and wearable platforms, but their electromechanical response remains modest. Hybrid materials—polymer matrices filled with ceramic particles—have emerged as a promising compromise, improving stress transfer and enhancing piezoelectric output while retaining flexibility. However, their performance is still limited by interfacial adhesion, dielectric breakdown, and the difficulty of achieving uniform particle dispersion.

Beyond these intrinsic material trade-offs, future printable piezoelectric systems must also meet practical requirements related to manufacturability and long-term deployment. Many emerging applications—wearable health monitoring, soft robotics, and distributed structural sensing—demand fabrication routes that are low-cost, solvent-efficient, and compatible with roll-to-roll or large-area processing. Reducing ink formulation complexity, minimizing thermal budgets, and enabling direct patterning without masks or multi-step lithography will be essential for scalable production. Equally important is long-term stability: printed piezoelectric devices must withstand humidity, mechanical fatigue, thermal cycling, and electrical depolarization over months or years of operation. Achieving this combination of convenient synthesis, low-cost processing, mechanical flexibility, and durability remains a central challenge for the field. The comparison also reveals a broader limitation in the field: reported d_33_ values are not always accompanied by sufficient information on measurement method, frequency, sample geometry, thickness, and poling history, making standardized testing and reporting essential for the future quantitative benchmarking of printed piezoelectric materials.

The comparative overview presented in Table 1 makes these contrasts explicit: ceramics dominate the upper end of d_33_ performance but demand extreme thermal budgets; polymers cluster at low temperatures with lower coefficients; hybrids populate the intermediate regime. This distribution reflects not only material limitations but also the absence of a unified framework for designing printable piezoelectric systems that combine high performance, low-temperature processing, and mechanical adaptability.

Looking ahead, several scientific directions appear poised to reshape the field. One of the most pressing challenges is the development of low-temperature routes to high-performance ceramics. Emerging strategies such as liquid-phase sintering, cold sintering, and surface functionalization of ceramic particles have already shown that it is possible to densify or activate ceramic structures without exposing them to extreme temperatures. If these approaches can be refined and generalized, they may enable ceramic-level performance on flexible substrates, textile platforms, and multi-material printing environments, opening the door to fully integrated wearable or implantable systems.

A unifying challenge across these directions is the absence of a predictive design framework that links ink formulation, processing conditions, microstructure evolution, and electromechanical response. Most current studies optimize one dimension—particle loading, β-phase content, sintering profile, or printing resolution—in isolation. A more rigorous, multiscale understanding of how processing history governs crystallinity, porosity, interfacial adhesion, and dielectric behavior would enable rational design rather than empirical tuning. Developing such a framework will require integrating rheological modeling, in situ characterization during printing, and microstructure-aware electromechanical simulations. This represents a critical opportunity for the field to transition from materials exploration to materials engineering.

Environmental considerations will also shape the next generation of printable piezoelectric materials. Lead-free ceramics such as BCZT, BaTiO_3_, and KNN have advanced significantly, but their performance still lags behind PZT. The continued exploration of dopants, grain boundary engineering, and compositional tuning will be essential for producing sustainable materials that do not compromise electromechanical sensitivity. In parallel, polymer chemistry offers opportunities to engineer new ferroelectric copolymers, block copolymer architectures, and supramolecular assemblies that could surpass the performance of conventional PVDF-based systems while retaining their mechanical advantages.

Long-term reliability is another underexplored but essential frontier. Printed piezoelectric materials—particularly polymer-based and hybrid systems—are susceptible to dielectric aging, moisture uptake, mechanical fatigue, and gradual depolarization under cyclic loading. For applications in wearables, robotics, and infrastructure monitoring, devices must maintain stable performance under repeated bending, stretching, and environmental exposure. Strategies such as encapsulation layers, hydrophobic surface treatments, fatigue-resistant polymer matrices, and thermally stable interfacial chemistries will be necessary to ensure operational lifetimes comparable to conventionally manufactured transducers.

Hybrid materials represent another fertile direction. Their performance depends critically on the interface between polymer matrices and ceramic fillers, and advances in surface chemistry—such as tailored coupling agents, engineered particle morphologies, and hierarchical filler architectures—could dramatically improve stress transfer and polarization efficiency.

As printing techniques evolve, hybrids may also benefit from spatially graded compositions, architected microstructures, and multi-phase designs that exploit the strengths of each constituent material.

Equally important is the refinement of printing techniques themselves. Each method—direct ink writing, stereolithography, projection micro-stereolithography, screen printing, and inkjet printing—offers distinct advantages, but all require inks with carefully tuned rheology, stability, and curing behavior. As researchers gain a deeper understanding of how ink formulation interacts with printing physics, it will become possible to fabricate increasingly intricate, high-resolution structures that exploit geometry as an active design parameter rather than a constraint. The convergence of materials design and geometric design is likely to be one of the defining themes of the next decade.

Cost and scalability will also determine whether printed piezoelectric technologies transition from laboratory demonstrations to commercial products. Many current formulations rely on high-purity ceramic powders, specialty solvents, or complex dispersants that increase cost and limit throughput. Future research should prioritize earth-abundant precursors, water-based or solvent-reduced inks, and printing workflows compatible with high-speed, roll-to-roll manufacturing. Simplifying ink chemistries while maintaining print fidelity and electromechanical performance will be key to enabling large-scale deployment in consumer electronics, medical devices, and industrial sensing.

Finally, the future of printable piezoelectric transducers will depend on how effectively they can be integrated into complete systems. Fully printed ultrasonic probes, wearable patches, soft robotic actuators, and self-powered sensing platforms are already beginning to appear.

As printing technologies mature, it will become feasible to fabricate not only the piezoelectric layer but also the electrodes, matching layers, backing materials, and housings in a single, unified workflow. Such integration will reduce assembly complexity, improve device reliability, and enable new classes of multifunctional, adaptive, and application-specific transducers.

Printable piezoelectric materials are entering a phase where incremental improvements in d_33_ or β-phase content are no longer sufficient. The next breakthroughs will come from integrating materials chemistry, interface engineering, geometric design, and scalable manufacturing into a coherent design philosophy. Achieving high performance at low processing temperatures, ensuring long-term stability under real-world conditions, and enabling cost-effective, large-area fabrication will determine which technologies mature into practical transducers. With coordinated advances across materials, processing, and system integration, printable piezoelectrics will be ready to transition from promising prototypes to foundational components in next-generation robotics, structural health monitoring, energy harvesting, and biomedical technologies.

Ultimately, the next major advances will come not from incremental improvements in individual materials, but from a more integrated understanding of how formulation, processing, microstructure, and device architecture interact to define electromechanical performance.

## Figures and Tables

**Table 1:** Comparison of reported processing conditions and piezoelectric response of printed and printable piezoelectric materials. Poling fields are standardized to kV mm^−1^. Reported d_33_ values are included when available, but direct cross-study comparison should be made cautiously because measurement method, geometry, and testing conditions are not standardized across the literature *.

Ref.	Printing Method	Piezoelectric Material	Sintering Temperature (°C)	Poling Field (kV/mm)	d_33_ (pC/N)	Proposed Applications
[61]	DIW	PZT	1250	2.5	103	underwater acoustic transducer
[62]	DIW	PZT	1250	3.5	265	-
[63]	MIP-SL	PZT	1100	3	583	ultrasonic transducers
[64]	DIW	PLZT	1200	3.5	347	-
[66]	DIW	BCZT	1500	3	100	-
[67]	DIW	KNN	1130	2.5	280	-
[68]	MIP-SL	BaTiO_3_	1330	3	160	ultrasonic transducer
[69]	MIP-SL	BaTiO_3_	1350	2	60	ultrasonic transducer
[70]	screen printing	BaTiO_3_	150	6	1.31	load sensor
[71]	MIP-SL	PZT	0	-	100	pressure sensor
[72]	spin coating	P(VDF-TrFE)	140	120	-	ultrasonic transducers
[73]	screen printing	P(VDF-TrFE)	100	80	32	pressure sensor
[74]	inkjet printing	P(VDF-TrFE)	140	100	10.4	pressure sensor, cantilever actuator, energy harvesting
[75]	stencil printing	P(VDF-TrFE)	120	65	22	pressure sensor
[76]	spray, screen printing, doctor blading	P(VDF-TrFE)	230	100	19	environmentally friendly pressure sensors
[77]	extrusion casting	PVDF	250	240	4	-
[77]	extrusion casting	PZT/PVDF	250	240	9	-
[77]	extrusion casting	PZT@105/PVDF	250	240	15	-
[77]	extrusion casting	PVDF/PZT@105/BNNS	250	240	21	-
[78]	doctor blading/wet coating	P(VDF-TrFE)	140	50	19.8–27.1	energy harvesting
[79]	DIW	PVDF/BaTiO_3_	120	50	20	energy harvesting
[80]	DIW	BCZT NW/P(VDF-TrFE)	70	10	30	piezoelectric nanogenerator, energy harvesting, sensing
[81]	screen printing	PVDF	80	50	16	flexible pressure sensor, PENG
[81]	screen printing	TOS- BaTiO_3_/PVDF	80	50	33.5	flexible pressure sensor, touch sensor, energy harvesting
[82]	DIW	PVDF/BaTiO_3_	180	-	-	energy harvesting
[83]	multi-extrusion	PVDF-HFP/BaTiO_3_	260	20	7.3	3D-printed piezoelectric sensors, pressure sensing
[84]	solvent casting	CLFO/PVP/ PVDF	120	-	-	energy harvesting
[85]	doctor blade/solution casting	M-phosphate/PVDF, M=Ni, Ag, Co	70	-	-	energy harvesting
[86]	Electric field-assisted DIW	PVDF/graphene	70	-	-	piezoelectric thin-film sensor, vibration sensing
[87]	solution casting	rGO/PVDF	90	-	39.3	piezoelectric energy transfer, ferroelectric storage
[88]	melt extrusion	PVDF/graphene	170–190	-	-	dielectric energy storage, sensors, microcapacitors
[89]	DIW	g-C_3_N_4_/PVDF	-	-	15.9	self-powered wearable sensors, IoT devices, energy harvesting
[90]	DIW	PVDF/ ILs@CNTs	180	-	30	energy harvesting, flexible sensor
[91]	ink deposition printing	LIG/P(VDF-TrFE)	140	170	23	flexible ultrasound transducer, wearable imaging
[92]	CO_2_-laser LIG/deposition	P(VDF-TrFE) w/LIG, LIG-Au, Au, Ag, or graphene	140	200	25	flexible ultrasound transducer, wearable/conformal imaging

* Note: Poling fields were standardized to kV mm^−1^ to enable direct comparison. Values originally reported in V µm^−1^, MV m^−1^, kV cm^−1^, or MV cm^−1^ were converted using equivalent field units. Entries marked “-“ indicate that the value was not reported in the original study or could not be converted reliably from the available information. The d_33_ values are reported as provided in the cited studies and should not be interpreted as fully normalized material constants, because measurement method, loading frequency, sample thickness, electrode geometry, active volume, substrate constraint, and poling time/temperature are not reported consistently across the literature.

## Data Availability

No new data were created.
